# The impact of COVID-19 on recovery in Henoch-Schönlein purpura patients: a cross-sectional questionnaire study during a distinctive period

**DOI:** 10.3389/fped.2025.1635822

**Published:** 2026-01-16

**Authors:** Lihua Jin, Caixia Zhao, Jiao Xiong, Qiqi Chang, Yan Su, Binjing Dou, Li Zhang, Ping He

**Affiliations:** 1 Department of Rehabilitation Medicine, Second Affiliated Hospital of Kunming Medical University, Kunming, Yunnan, China; 2 Department of Pediatrics, Yunnan University of Traditional Chinese Medicine, Kunming, Yunnan, China; 3 Department of Pediatrics, First Affiliated Hospital of Yunnan University of Traditional Chinese Medicine, Kunming, Yunnan, China

**Keywords:** Henoch-Schönlein purpura, COVID-19, vaccination, pediatrics, propensity score matching

## Abstract

**Background:**

The rising incidence of infectious diseases underscores the necessity for vaccination such as COVID-19. Beyond examining the side effects in healthy individuals, it is crucial to investigate the vaccination experiences of vulnerable populations, particularly those with Henoch-Schönlein purpura (HSP).

**Methods:**

A questionnaire study was conducted during the period of rapid outbreak following the relaxation of travel restrictions in China towards the latter stages of the COVID-19 pandemic.

**Results:**

Despite non-HSP individuals exhibiting more pronounced symptoms of cough, fatigue, dizziness, and headache compared to HSP patients, the HSP group displayed significantly lower rates of vaccination post-matching. Specifically, only 63% of HSP individuals completed the full vaccination regimen, with no significant association found between vaccination status and improved recovery or mitigation of HSP symptoms. Among HSP individuals, only improvement in diarrhea symptoms was positively correlated with recovery time, while fully vaccinated HSP children exhibited more abnormal symptoms during the recovery period.

**Conclusion:**

Results from this study on COVID-19 vaccination status among pediatric patients who required hospital visits during the peak of the pandemic indicated that vaccination rates were comparatively lower among patients with HSP, even amidst severe outbreaks. HSP patients who completed the full vaccination regimen appeared to experience more pronounced adverse symptoms. This observation suggests that their increased vaccine hesitancy relative to the general population may be justified and warrants careful consideration.

## 1 Introduction

The rising incidence of infectious diseases underscores the critical need for vaccination as a means to control viral transmission (1). The vaccination strategies implemented during the COVID-19 pandemic underscore the pivotal role of preventive measures in epidemic management. Despite inherent disparities in vaccination rates across different populations, these variations can amplify health risks during disease outbreaks (2). As of December 23, 2022, China had administered 3.46 billion doses of COVID-19 vaccines to its population of 1.4 billion (3), underscoring the prevailing consensus that the benefits of vaccination generally surpass the associated risks. Nonetheless, certain academic studies have reported instances of Henoch-Schönlein Purpura (HSP) subsequent to COVID-19 infection (4, 5). Casini et al. (6) have documented occurrences of heat shock syndrome following vaccination, a phenomenon not uncommon, given that infections, including those induced by vaccination, are known to potentially trigger HSP. The impact of vaccination on patients with pre-existing HSP and the potential for an increased incidence of adverse effects post-vaccination require further investigation. It is crucial for healthcare administrators to remain cognizant of these issues.

While the catastrophic phase of the pandemic having passed, and vaccination is currently not compulsory, vulnerable individuals present a societal concern in the context of mandatory vaccination. Mandatory vaccination can be regarded as a protective strategy for safeguarding vulnerable populations against preventable diseases (7). Nonetheless, it may also intensify the psychological burden on individuals who are medically contraindicated for vaccination due to some side effect (8, 9). Prior research has indicated that COVID-19 infection or vaccination may have a heightened effect on glomerular diseases, specifically increasing the risk of HSP (10).Then, what is the prognosis for children with a previous history of HSP following infection with COVID-19? Would they have a lower vaccination rate due to concerns or would their recovery be worse due to the virus infection? Based on current information, there is a lack of relevant data available. It is imperative to promptly gain an understanding of their outcomes post-illness.

China has experienced multiple waves of COVID-19 outbreaks, with a significant surge in cases occurring in December 2022 following the lifting of lockdown measures in Guangdong Province, resulting in heightened strain on respiratory and pediatric healthcare facilities (11). During the period leading up to January 2023, certain Class 3A hospitals in China's southwest region were compelled to temporarily function as primary care providers in order to accommodate the influx of patients with respiratory infections. This study aims to investigate the vaccination status of families with individuals diagnosed with HSP who sought treatment at pediatric/adolescent clinics during this special stime frame, and to assess potential disparities in recovery outcomes between these individuals and non-HSP patients.

Our hypothesis posits that individuals with HSP may exhibit a greater rate of respiratory symptoms and comorbidities during the recovery phase in comparison to their non-HSP counterparts. Additionally, it is plausible that HSP pediatric/adolescent may have a higher rate of COVID-19 vaccination uptake, potentially leading to an exacerbation of their HSP symptoms upon contracting the virus.

## 2 Methods

### 2.1 Design

This cross-sectional questionnaire study was conducted from December 20, 2022 to January 20, 2023, coinciding with the peak of the epidemic in the region. The questionnaire method was utilized for pediatric/adolescent outpatients (include the HSP clinic and normal clinic), with the survey being conducted online after scanning a QR code to minimize contact. Following the inclusion criteria and completion of the consultation, family members of the patients were invited to participate in the study and provided with an explanation of its purpose. Participants accessed and submitted the questionnaire survey through the WeChat app by scanning a code with their mobile phones online.

It is important to emphasize that participation in the study is entirely voluntary, with patient information being kept confidential. Individuals who opt not to participate may disregard the invitation. This research adhered to the principles outlined in the Declaration of Helsinki and was approved by the Institutional Review Board (IRB) of the Affiliated Hospital of Yunnan Province Traditional Chinese Medicine University.

### 2.2 Participants

Individuals under the age of 18 who had a documented history of COVID-19 infection, with or without a diagnosis of HSP, and who had attended the outpatient department of our center between December 20, 2022, and January 20, 2023, were included in the study. After positive test, inclusion criteria required a negative COVID-19 test result within 60 days prior to the investigation date, and determined to be in the recovery period, absence of significant cognitive impairment in both the individuals and their guardian, ability to comprehend and communicate physical discomfort, and willingness to participate in the research.

The specific types of vaccines utilized were not examined in this study, as they were distributed at no cost by the national government amidst the pandemic. Furthermore, it is noteworthy that only inactivated vaccines were accessible in mainland China during this timeframe.

### 2.3 Measurements

The survey assessed demographic attributes and immunization status (having received at least two vaccinations or no more than one vaccination for personal or other reasons). These metrics were refined and formulated by a committee comprising four pediatric faculty members of associate professor rank or higher. In the absence of existing publicly accessible surveys pertaining to the recuperation of children from COVID-19 infection, a questionnaire was designed to assess the persistent symptoms experienced by both undiagnosed and diagnosed HSP children throughout their COVID-19 recovery process up until the day of data collection, ensuring a rigorous and standardized approach for comparative analysis. The original questionnaires comprised 11 and 22 items, respectively. Following evaluation by an expert panel, two items were eliminated, and the remaining items were revised to facilitate straightforward and prompt responses aligned with the research goals. Each item allowed for a binary response of “Yes” or “No”, with the exception of the query regarding the perceived exacerbation of HSP symptoms post-COVID-19, which included an additional response option of “Unclear”. The primary aims of this study were to examine potential disparities in the recovery trajectory between pediatric patients with and without a diagnosis of HSP following COVID-19, as well as to explore potential variations in symptoms or manifestations during recovery among vaccinated children with HSP. Consequently, the questionnaire items were designed to elicit binary responses in order to facilitate intergroup comparative analysis. The symptoms under investigation primarily concern those that have persisted following a negative SARS-CoV-2 test result. Symptomatology that resolved during the interim period after a confirmed negative COVID-19 diagnosis should not be marked as “Yes”. The questionnaire could be found at the *Figshare* (doi: 10.6084/m9.*figshare*.25605228).

### 2.4 Data analysis

Since all variables were non-parametric, the Mann–Whitney *U* test was used to compare the quantitative data between the two groups, while qualitative variables were compared using contingency tables, Fisher's exact test, or Linear-by-Linear Association. These methods were employed to compare the general conditions and post-COVID-19 recovery situations between the two groups. Given the differences in age and disease course between the baseline groups, we employed the Propensity score matching (PSM) method to select children with Nor who were matched in age and disease course to those in the HSP group, and conducted a renewed comparison between the groups. PSM was used to minimize the potential confounding effects of age and duration. The HSP group children were chosen as the study group to observe the differences in the recovery process of undiagnosed HSP children and diagnosed HSP children after COVID-19. SPSS software was employed for PSM, with age and duration set as the predictors, and a match tolerance of 0.003 was established due to the minimal difference in scores leading to either smaller or statistically significant differences between the Nor group children. A subgroup analysis was performed on the HSP group children, comparing the effects of vaccination on recovery and whether it exacerbated HSP symptoms during the COVID-19 infection process. Lastly, a Spearman correlation analysis was conducted on the general conditions and recovery-related symptoms of the HSP group children to assess their correlations. All statistical analyses were performed using SPSS v 26 (IBM Corp., Armonk, NY, USA); statistical significance was set as at *p* ≤ 0.05.

## 3 Results

A total of 80 children with HSP and 48 children without HSP were initially enrolled in the study. However, due to notable variations in age (*p* = 0.000) and duration after negative of COVID-19 infection test (*p* = 0.027) between the two groups, PSM adjustment was product and matching the 28 children from the non-HSP group for comparative analysis. Prior to adjustment, disparities were observed between the two groups in terms of symptoms such as expectoration (*p* < 0.001), fatigue (*p* < 0.001), and dizziness or headache (*p* < 0.001) during the recovery phase. Following adjustment, disparities persisted between the two groups in the initial three symptoms (*p* < 0.05), suggesting potential distinctions in the recovery process following COVID-19 infection among children with and without HSP. Furthermore, variations in vaccination status were also observed between the groups. While the HSP cohort exhibited a lower likelihood of being unvaccinated prior to propensity score matching, this tendency notably escalated post-adjustment, exceeding that of the non-HSP children (*p* = 0.003, Table 1).

**Table 1:** Demographic characteristics of participants before PSM matching and after.

Items	Un-matched			Matched		
	HSP group	Nor GROUP	*P* value	HSP group	Nor GROUP	*p*-value
	(*n* = 80)	(*n* = 48)		(*n* = 80)	(*n* = 28)	
Age (years)	10.0 (0.8–17)	6.0 (0.5–16)	**0.000** *	10.0 (0.8–17)	9.0 (4–13)	0.130
Duration after negative COVID-19 test (days)	15 (2–59)	18 (4–54)	**0.027** *	15 (2–59)	12 (5–27)	0.249
Gender						
Male	37 (46.25%)	25 (52.08%)	0.523	37 (46.25%)	10 (35.71%)	0.333
Female	43 (53.75%)	23 (47.92%)		43 (53.75%)	18 (64.29%)	
Vaccination						
Vaccinated (more than twice)	51 (63.75%)	38 (79.17%)	0.067	51 (63.75%)	26 (92.86%)	**0.003** *
Unvaccinated (once or never)	29 (36.25%)	10 (20.83%)		29 (36.25%)	2 (7.14%)	
During recovery period						
Cough						
Yes	55 (68.75%)	29 (60.42%)	0.337	55 (68.75%)	16 (57.14%)	0.265
No	25 (31.25%)	19 (39.58%)		25 (31.25%)	12 (42.86%)	
Expectoration						
Yes	47 (58.75%)	9 (18.75%)	**<0.001** *	47 (58.75%)	8 (28.57%)	**0.006** *
No	33 (41.25%)	39 (81.25%)		33 (41.25%)	20 (71.43%)	
Congestion						
Yes	39 (48.75%)	15 (31.25%)	0.052	39 (48.75%)	10 (35.71%)	0.233
No	41 (51.25%)	33 (68.75%)		41 (51.25%)	18 (64.29%)	
Rhinorrhea						
Yes	31 (38.75%)	12 (25%)	0.111	31 (38.75%)	10 (35.71%)	0.776
No	49 (61.25%)	36 (75%)		49 (61.25%)	18 (64.29%)	
Fatigue						
Yes	38 (47.50%)	6 (12.50%)	**<0.001** *	38 (47.50%)	2 (7.14%)	**<0.001** *
No	42 (52.50%)	42 (87.50%)		42 (52.50%)	26 (92.86%)	
Taste alteration						
Yes	20 (25%)	9 (18.75%)	0.413	20 (25%)	8 (28.57%)	0.711
No	60 (75%)	39 (81.25%)		60 (75%)	20 (71.43%)	
Olfactory alteration						
Yes	13 (16.25%)	8 (16.66%)	0.951	13 (16.25%)	8 (28.57%)	0.156
No	67 (83.75%)	40 (83.33%)		67 (83.75%)	20 (71.43%)	
Diarrhea						
Yes	10 (12.50%)	3 (6.25%)	0.368	10 (12.50%)	0 (0%)	0.061
No	70 (87.50%)	45 (93.75%)		70 (87.50%)	28 (100%)	
Dizziness or headache						
Yes	50 (62.50%)	7 (14.58%)	**<0.001** *	50(62.50%)	6(21.43%)	**<0.001** *
No	30 (37.50%)	41 (85.42%)		30 (37.50%)	22 (78.57%)	

Values are expressed as the percentage (%) or median (range).

PSM, propensity score matching; HSP, Henoch-Schönlein purpura.

* *p*-values were calculated using Mann–Whitney *U* test, Pearson Chi-Square, or Fisher's Exact Test.

*p-values were calculated using the Mann-Whitney *U* test.

We conducted a comparative analysis of recovery period symptoms among HSP children who had received vaccination (more than twice) or unvaccination (once or never). Our findings revealed no statistically significant disparities in respiratory symptoms, skin rash recurrence, and other symptoms (*p* > 0.05). Nevertheless, it appeared that children who had completed the full vaccination course exhibited a higher prevalence of abnormal symptoms (Table 2).

**Table 2:** Different symptoms during the recovery period between vaccinated and unvaccinated in HSP children.

Items	Vaccinated (more than twice)	Unvaccinated (once or never)	*p*-value
Age (year)	9 (4.6–17)	11 (0.8–17)	0.920
Duration after negative COVID-19 test (days)	15 (1–58)	15 (2–28)	0.389
Gender			
Male	21 (41.18%)	16 (55.17%)	0.227
Female	30 (58.82%)	13 (44.83%)	
During recovery period			
Cough			
Yes	36 (70.59%)	19 (65.52%)	0.638
No	15 (29.41%)	10 (34.48%)	
Expectoration			
Yes	33 (64.71%)	14 (48.28%)	0.151
No	18 (35.29%)	15 (51.72%)	
Congestion			
Yes	26 (50.98%)	13 (44.83%)	0.597
No	25 (49.02%)	16 (55.17%)	
Rhinorrhea			
Yes	22 (43.14%)	9 (31.03%)	0.285
No	29 (56.86%)	20 (68.97%)	
Fatigue			
Yes	27 (52.94%)	11 (37.93%)	0.196
No	24 (47.06%)	18 (62.07%)	
Taste alteration			
Yes	13 (25.49%)	7 (24.14%)	0.893
No	38 (74.51%)	22 (75.86%)	
Olfactory alteration			
Yes	7 (13.73%)	6 (20.69%)	0.417
No	44 (86.27%)	23 (79.31%)	
Diarrhea			
Yes	6 (11.76%)	4 (13.79%)	1.000
No	45 (88.24%)	25 (86.21%)	
Dizziness			
Yes	31 (60.78%)	19 (65.52%)	0.674
No	20 (39.22%)	10 (34.48%)	
Headache			
Yes	30 (58.82%)	20 (68.97%)	0.368
No	21 (41.18%)	9 (31.03%)	
Pharyngitis			
Yes	22 (43.14%)	11 (37.93%)	0.649
No	29 (56.86%)	18 (62.07%)	
Pharyngeal itching and dryness			
Yes	24 (47.06%)	12 (41.38%)	0.624
No	27 (52.94%)	17 (58.62%)	
Hoarseness			
Yes	11 (21.57%)	8 (27.59%)	0.543
No	40 (78.43%)	21 (72.41%)	
Body pain			
Yes	17 (33.33%)	6 (20.69%)	0.230
No	34 (66.67%)	23 (79.31%)	
Skin rash			
Yes	39 (76.47%)	21 (72.41%)	0.687
No	12 (23.53%)	8 (27.59%)	
Gastrointestinal symptoms			
Yes	44 (86.27%)	26 (89.66%)	0.740
No	7 (13.73%)	3 (10.34%)	
Joint pain			
Yes	46 (90.2%)	24 (82.76%)	0.334
No	5 (9.8%)	5 (17.24%)	
Kidney manifestations			
Yes	43 (84.31%)	25 (86.21%)	0.820
No	8 (15.69%)	4 (13.79%)	
Exacerbation or recurrence of HSP			
Yes	7 (13.73%)	4 (13.79%)	0.971
No	35 (68.63%)	20 (68.97%)	
Unclear	9 (17.65%)	5 (17.24%)	

Values are expressed as the percentage (%) or median (range).

HSP, Henoch-Schönlein purpura.

Children diagnosed with HSP exhibit a similar proportion of cases in which symptoms worsen following infection with COVID-19, regardless of vaccination status. Specifically, 13.73% and 13.79% of vaccinated and unvaccinated HSP patients, respectively, experienced exacerbation of symptoms, with no statistically significant difference observed (Table 2). Additionally, among the 48 non-HSP children included in the study, one child was diagnosed with HSP as a result of COVID-19 infection, representing 2.08% of this subgroup although not shown.

**Figure 1:**
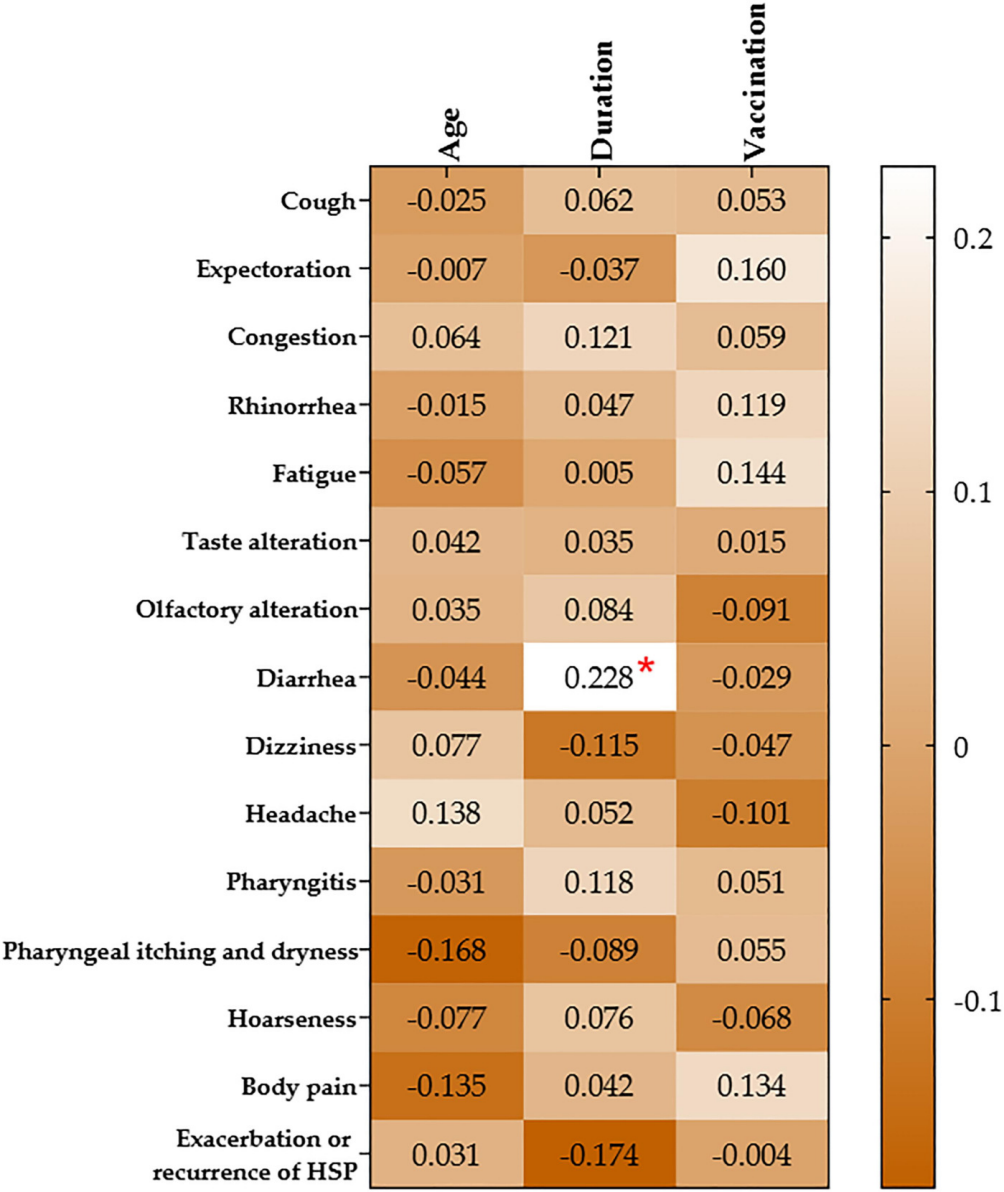
Correlation between symptoms in recovery period and demographics in HSP children. *p-values were calculated using Spearman correlation analysis. HSP, Henoch-Schönlein purpura.

We endeavored to analyze the age, disease progression post-COVID-19 infection, and vaccination status of children with HSP in relation to variations in symptoms during the recovery period. Our findings indicate a positive correlation between the duration of the recovery period and a decrease in the occurrence of diarrhea symptoms among HSP children (*r* = 0.228, *p* = 0.042). Conversely, no significant correlations were observed between the length of the recovery period and other symptoms (*p* > 0.05, Figure 1).

In patients without a confirmed diagnosis of HSP, there is no significant correlation between duration after COVID-19 infection, age, vaccination history, and the presence of symptoms during the recovery phase, both pre- and post-adjustment (*p* > 0.05).

## 4 Discussion

Since the onset of the COVID-19 pandemic in 2019, there has been a marked increase in research publications examining the vaccines (12). While prior studies have predominantly concentrated on vaccine safety, particularly concerning heat shock in the context of severe cardiovascular events (13), there appears to be a relative paucity of attention, compared to broader academic discourse, on the global impact of COVID-19 on patients with pre-existing heat shock conditions.

The predominant trigger factor for HSP is a preceding upper respiratory tract infection, with viral infections, particularly human parvovirus B19 (14), being the most common pathogenic factor. Given these factors, it is plausible that COVID-19 could exacerbate or precipitate HSP in affected individuals. Our study aimed to assess the potential impact of COVID-19, a disease that has swept across the globe and is still ongoing, on children already diagnosed with HSP. Especially about the potential increased susceptibility of children with preexisting HSP to COVID-19, specifically in relation to respiratory symptoms during recovery period and whether exacerbation of HSP symptoms due to COVID-19. Contrary to our initial hypothesis, the findings did not support this association.

In our research, it was observed that symptoms such as expectoration, fatigue, dizziness, and headache appeared to be more common in HSP patients, regardless of adjustments made for age and post-viral infection course. The observed phenomena may be attributed to the immune response elicited by the vaccine itself, as vaccination has the potential to trigger or exacerbate certain immune-related conditions (15). Furthermore, it could be linked to the extended administration of oral corticosteroids in pediatric patients with HSP, which may result in dysbiosis of the gut microbiota (16). Given the pivotal role of the gut microbiota in immune modulation (17), this dysbiosis may be a primary factor contributing to the prolonged abnormal symptoms observed in children with HSP compared to their non-HSP counterparts. The incidence of diarrhea in HSP children within our study, which may influence the persistence of symptoms, further corroborates the hypothesis that gut microbiota dysbiosis, leading to immune abnormalities, might play a role in the sustained abnormal symptoms in HSP children. Nonetheless, our research has not yet explored this area, and it merits further investigation in future studies.

The unexpectedly low vaccination rate among children with HSP in this study contrasts with the country's generally high vaccination and acceptance rates. Despite China's implementation of measures such as the “health code” system to restrict travel for unvaccinated individuals (18), only 63% of HSP individuals in our study have received the recommended three doses of the vaccine, a relatively rare occurrence in a country with typically high vaccination compliance.

This phenomenon reflects societal issues regarding public trust and compliance with immunization programs, specifically highlighting the influence of media coverage on vaccination efforts. This is evident in the context of vaccine accessibility during pandemics, which may have significant implications for public health and safety, particularly for vulnerable populations (19). During the initial stages of the pandemic, a significant proportion of the Chinese population displayed a strong willingness to receive COVID-19 vaccinations. Nevertheless, over time, this enthusiasm waned, particularly among parents of children with HSP. It has been postulated that a minimum of 67% of a fully susceptible populace must be immunized to attain herd immunity (20). However, even the implementation of travel restrictions by the government failed to assuage their apprehensions regarding the vaccine, especially within the subset of parents with children affected by HSP. These concerns have been further heightened by the growing number of reports indicating exacerbation of HSP following either COVID-19 infection or vaccination, especially in the later stages of the pandemic (21). It is evident that a significant portion of HSP caregivers in our study were adversely affected by the negative media coverage surrounding this issue.

Therefore, we endeavored to analyze the age, disease progression post-COVID-19 infection, and vaccination status of children with HSP in relation to variations in symptoms during the recovery period. Our findings indicate a positive correlation between the duration of the recovery period and a decrease in the occurrence of diarrhea symptoms among HSP children. Conversely, no significant correlations were observed between the length of the recovery period and other symptoms.

Initially, we expected that vaccinated individuals with HSP would display a decrease in atypical symptoms associated with respiratory issues or post-vaccination recuperation. It was theorized that these symptoms would decrease over time as recovery progressed. Nevertheless, our research uncovered a potentially elevated occurrence of unusual sensations in children with hypersensitivity pneumonitis who had completed the full vaccination series. While this discovery lacks statistical significance and necessitates additional validation through larger sample sizes, it does prompt a more cautious approach when considering vaccination for this population.

It is imperative to acknowledge that our survey did not include the psychologic status of parents, whose increased awareness stemming from their child's condition may have introduced recall bias, as they may be more vigilant in detecting any anomalies compared to parents who have the luxury of opting out of vaccination. Nevertheless, these findings necessitate further empirical research for confirmation. Our study indicates a lower rate of abnormal symptoms in the HSP cohort compared to non-HSP participants at baseline, suggesting a superior recovery rate in HSP children during the observation period. However, it is important to note that our analysis does not include HSP patients who required hospitalization due to severe exacerbation following infection, limiting the generalizability of our findings. Therefore, while our results suggest a positive outcome for HSP, further research is needed to fully understand the implications of these findings.

During the period following the lifting of lockdown restrictions amidst heightened pandemic activity, we conducted an analysis of the recovery trajectories of pediatric/adolescents' outpatients in specialized clinic for HSP and general clinic in a general hospital following infection with COVID-19. It is noteworthy that, despite a comparatively low rate of vaccination among HSP patients, there was no statistically significant variance in the incidence of HSP exacerbations linked to the virus between vaccinated and unvaccinated individuals. Interestingly, the patients with a confirmed diagnosis of HSP in the cohort displayed superior recovery outcomes compared to those without such a diagnosis. Additionally, our results suggest that HSP children who had received the full vaccine regimen showed a higher incidence of lingering symptoms during the recovery period in comparison to those who had not completed their vaccinations.

## Data Availability

The original contributions presented in the study are included in the article/Supplementary Material, further inquiries can be directed to the corresponding author/s.
